# Supplementary material for: Effects of Probiotics Administration on Human Metabolic Phenotype
Source: Metabolites. 2020 Oct 7;10(10):396. doi: 10.3390/metabo10100396 (PMC7601401; doi:10.3390/metabo10100396)
Supplement: Supplementary file 1 [file metabolites-10-00396-s001.pdf]

Supporting Material

Effects of Probiotics Administration on Human Metabolic Phenotype

Veronica Ghini[1], Leonardo Tenori[2,3], Marco Pane[4], Angela Amoruso[4], Giada Marroncini[5], Diletta Francesca Squarzanti[6,7], Barbara Azzimonti[6,7], Roberta Rolla[6,8], Paola Savoia[6,9], Mirko Tarocchi[5], Andrea Galli[5], Claudio Luchinat*[1,2,3].

1 Consorzio Interuniversitario Risonanze Magnetiche di Metallo Proteine (CIRMMP), Sesto Fiorentino, Italy.
2 Magnetic Resonance Center (CERM), University of Florence, Sesto Fiorentino, Italy.
3 Department of Chemistry, University of Florence, Sesto Fiorentino, Italy.
4 Probiotical S.p.A., Novara, Italy.
5 Department of Experimental and Clinical Biochemical Sciences "Mario Serio", University of Florence, Firenze, Italy.
6 Department of Health Sciences (DiSS), University of Piemonte Orientale (UPO), Via Solaroli 17, 28100 Novara, Italy.
7 Center for Translational Research on Autoimmune and Allergic Diseases (CAAD), DiSS, UPO, Corso Trieste 15/A, 28100 Novara, Italy.
8 Clinical Chemistry Unit, Azienda Ospedaliero Universitaria Maggiore della Carità, Corso Mazzini 18, 28100, Novara, Italy
9 SCDU Dermatology, AOU Maggiore della Carità, Novara, Italy.

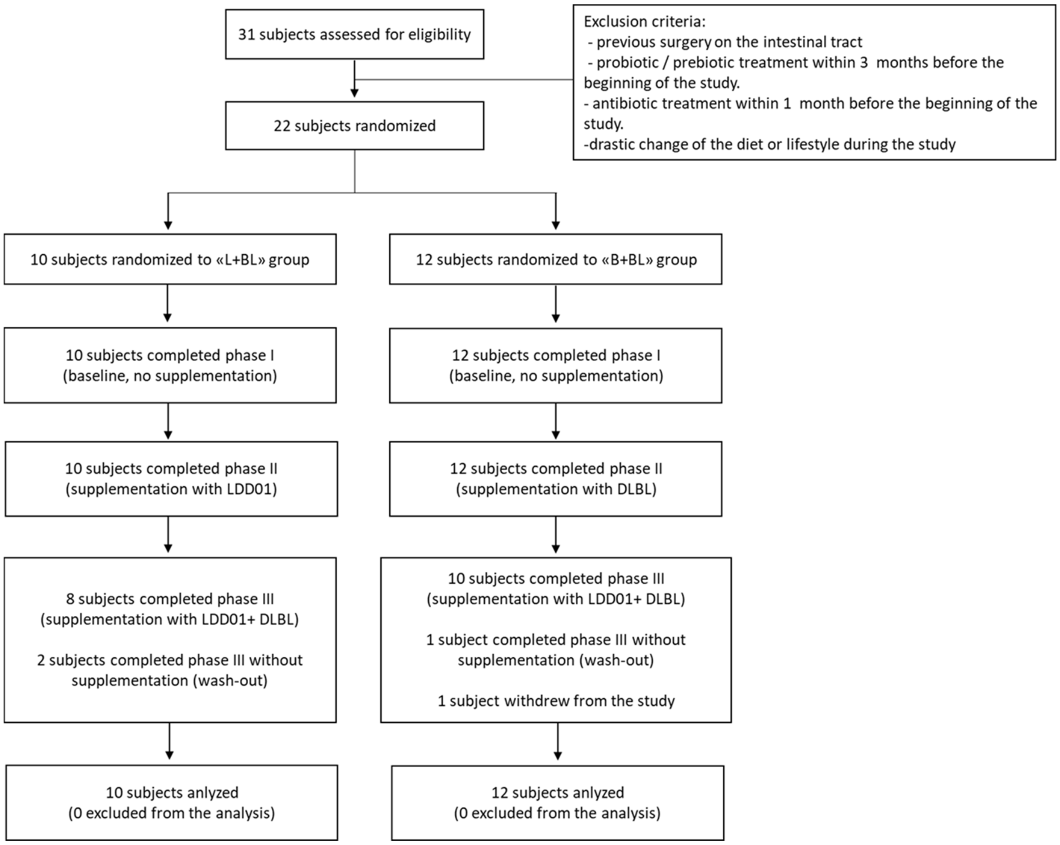

Figure S1. Paticipantion Flowchart.
